# Disentangling the Impact of Artistic Creativity on Creative Thinking, Working Memory, Attention, and Intelligence: Evidence for Domain-Specific Relationships with a New Self-Report Questionnaire

**DOI:** 10.3389/fpsyg.2016.01089

**Published:** 2016-07-28

**Authors:** Katrin Lunke, Beat Meier

**Affiliations:** Institute of Psychology and Center for Cognition, Learning and Memory, University of BernBern, Switzerland

**Keywords:** artistic creativity, divergent thinking, convergent thinking, creativity questionnaire, intelligence

## Abstract

The goal of the present study was to take a new look at the relationship between creativity and cognitive functioning. Based on models that have postulated domain- and sub-domain-structures for different forms of creativity, like scientific, technical or artistic creativity with cognitive functions as important basis, we developed a new questionnaire. The Artistic Creativity Domains Compendium (ACDC) assesses interest, ability and performance in a distinct way for different domains of artistic creativity. We present the data of 270 adults tested with the ACDC, standard tests of divergent and convergent thinking, and tests of cognitive functions. We present fine-grained analyses on the internal and external validity of the ACDC and on the relationships between creativity, working memory, attention, and intelligence. Our results indicate domain-specific associations between creativity and attention as well as working memory. We conclude that the ACDC is a valid instrument to assess artistic creativity and that a fine-grained analysis reveals distinct patterns of relationships between separate domains of creativity and cognition.

## Introduction

“Creativity is intelligence having fun” says a quote alleged to Albert Einstein, suggesting that creativity and cognition are closely linked together. Often in contemporary research, however, the relationship between creativity and intelligence has been discussed controversially. While some researchers have distinguished the constructs from each other, others have described them as complements (Guilford, 1959; Wallach and Kogan, 1965; Hocevar, 1980; Runco and Chand, 1995; Sternberg and Lubart, 1999; Kaufman and Baer, 2005; Kaufman, 2012). Different domains and sub-domains of creativity and different levels of involvement into creative activities can be distinguished. Here, we introduce the *Artistic Creativity Domains Compendium* (ACDC), a new self-report-measure for artistic creativity. It separately assesses the four domains *visual arts, literature, music* and *performing arts* and 18 according sub-domains such as *painting, ballet-dancing* or *acting* on three levels of involvement, that is, *interest, ability*, and *performance*. We present data of a norm-sample of 270 adults and relate artistic creativity to measures of divergent and convergent thinking, working memory, attention, and intelligence.

Creativity can be defined as the ability to generate new and adaptive ideas or novel solutions to problems and it is thus considered as fundamental for human civilization (Sternberg and Lubart, 1999; Takeuchi et al., 2011). It can be divided into divergent and convergent thinking and is usually tested by verbal or figural output (e.g., Mednick and Mednick, 1971; Goff and Torrance, 2002). Divergent thinking is characterized by the production of many different original solutions – rather than only one; convergent thinking is characterized by finding the one and only correct solution for a given problem. Additionally, self-report and third-person-questionnaires can be used to assess creativity in terms of creative operations, achievements, and creative activities (e.g., Hocevar, 1980; Carson et al., 2005; Antonietti et al., 2011; Kaufman, 2012).

Beyond the general definition, creativity is a versatile construct that can be expressed in many forms, domains and facets. Artistic, scientific, and technical creativity have been proposed as specific forms in previous research (Stein, 1953; Davis et al., 2011; Kaufman, 2012; Kozhevnikov et al., 2013). Moreover, each different form of creativity includes different domains and these yet sub-domains, such as design, scriptwriting and crafting (e.g., Carson et al., 2005; Kaufman, 2012; Glaveanu et al., 2013). Due to the complexity of specific forms of creativity, the definition what a new and adaptive product should be like and how it is created has to be further specified. Next, we describe three key points determining artistic creativity (independence of time pressure, domain-width, and levels of involvement) and how we propose to assess them.

First, as the process of artistic creativity is likely to be more time-consuming than stimulus triggered divergent or convergent thinking due to domain specific stages, self-report questionnaires provide the opportunity to report past achievements specific for artistic creativity, instead of pressing the participants to produce creative solutions under non-ecological time pressure. Thus, we decided to develop a questionnaire that protocols artistic creativity completely independent of time pressure.

Second, artistic creative thinking is not limited to figural and verbal modes of expression. While painting, sculpting and designing can be easily described as figural expressions, and writing certainly is a verbal form of expression, singing, dancing, and acting cannot be sufficiently characterized by only these two modes. Thus, it is important to cover a wide range of different domains and sub-domains as proposed by Carson et al. (2005) and Kaufman and Baer (2005). This offers a better perspective on different modes of artistic expression and also enables the analysis of specific relationships between different domains of artistic creativity and constructs of cognitive functioning (Davis et al., 2011; Kaufman, 2012). Moreover, different forms and domains of creativity are linked to different thinking styles and even different characteristics of intelligence (Carson et al., 2005; cf. Kaufman, 2012).

To take this into account, the ACDC addresses the four main artistic domains of *visual arts, literature, music* and *performing arts* separately. Moreover, for each domain related sub-domains are included. For *visual arts*, they include *painting, sculpting, photography*, and *graphic design*; for *literature*, they include *fictional writing, poetry, play writing*, and *journalism*; for *music* they include, *classical music, jazz music, rock music*, and *folk music* and for *performing arts*, they include *dancing, ballet*, and *acting in movies, theaters* and *musicals*. Thus, it is possible to investigate the relationships among the several domains, sub-domains and their superordinate modes of creative expression and differences in their relationships to cognitive functions with the ACDC.

Third, one can be interested in different domains, in each domain the level of ability can vary, and making the creative achievement available for others reflects creative performance. That is, the quality of artistic creative products is influenced by knowledge and technical expertise. Beyond coming up with creative ideas, creativity also involves the creation of an artistic output expressed in a specific domain. The individual either has the aim and ability to do so or not. If the ability is present, the production on a subsequent level can be more or less skilled. Ideally, it will then be judged in an appropriate frame of reference (Stein, 1953; Mulvenna, 2013). Levels of involvement in different creative domains have already been addressed in the Creative Achievements Questionnaire (CAQ; Carson et al., 2005), which includes several hierarchically organized levels of involvement. They presume that scores on lower levels are required to score on higher levels of achievement. In contrast, we suggest that the levels of involvement are not necessarily dependent and participants can score in any pattern. Therefore, the ACDC is organized in three levels of involvement: *interest, ability*, and *performance.* These can be assessed independently. The first level refers to the mere interest in a domain and sub-domain. The second level refers to past completion of creative accomplishments. The third level refers to publication of completed artworks. This differentiation of levels of involvement enables to assess the specificity of a certain degree of involvement for each (sub-) domain separately. It also provides for the possibility to assess the development of creative profiles over time. This may be particularly useful to assess progresses in development, to test the effectiveness of creativity training, and for the assessment of pathological changes related to neurodegenerative and/or psychological disorders (Flaherty, 2005; Inzelberg, 2013; see **Image 1** in the Supplementary Materials for a specific example of a personal profile). In sum, the ACDC is a self-report questionnaire to assess artistic creativity with its domains and subdomains on three levels of involvement (for the full questionnaire see **Table S1** in the Supplementary Materials).

In differentiating several forms and levels of involvement of artistic creativity, it is an interesting question how they further relate to cognitive functions. For divergent and convergent creativity, contradictory results between creativity and cognitive functioning have been found which have been explained as a function of overlaps in the assessment of the different constructs (Hocevar, 1980; Batey and Furnham, 2006). Kaufman and Baer (2005) explain creativity as a result of motivation and cognitive functioning, thus implying that different abilities may cause creativity in different domains. Similarly, Damasio (2001) proposed that different cognitive abilities relate to different forms, domains and sub-domains of creativity.

Several studies support this position. For example, Hocevar (1980) found that verbal intelligence was the best predictor of creativity in the literature-domain. Kaufman and Baer (2004) showed that different cognitive characteristics were related to different forms of creativity. Specifically, creativity in communication and writing correlated positively with verbal SAT scores whereas creativity in math correlated positively with mathematical SAT scores. Furnham and Crump (2013) found that art students scored higher on vigilance and in a verbal abstract reasoning task. Moreover, science students scored higher on an intelligence test as well as on a logical reasoning task and a numerical reasoning task. In addition, several studies found that physical activity had a positive influence on executive functioning such as attention and working memory (Best, 2010). As some domains and sub-domains of creativity include more or less physical activity, different relations for different domains are plausible. We consider it important to further specify the relationship between cognitive functioning and specific domains and sub-domains of artistic creativity and to compare the results with different behavioral creativity measures.

The first goal of this study was to validate a new self-report-questionnaire that assesses artistic creativity. The ACDC covers *interest, ability*, and *performance* in artistic domains and sub-domains of different modes of creative expression. The second goal was to test the relationship between artistic creativity and common tests of figural and verbal, divergent and convergent thinking, in order to test the external validity of the ACDC. The third goal was to relate artistic creativity and divergent and convergent thinking to cognitive functions such as working memory, attention, and intelligence. This enables us to generate new insights into the relationships between domain-specific artistic creativity, typical measures of creativity (such as convergent and divergent thinking) and cognitive functions more generally.

In line with the suggestion that creativity is domain-specific (e.g., Kaufman and Baer, 2005), we expected specific relationships between divergent and convergent creativity and the ACDC for figural and verbal domains of artistic creativity, such as painting or writing. Moreover, in line with the suggestion that overlap in processing requirements determines the relationship between creativity and cognitive functions (e.g., Hocevar, 1980), we expected that cognitive functions would be associated differently for the different domains, sub-domains and levels of involvement of artistic creativity.

## Materials and Methods

### Scale Construction of the ACDC

The ACDC includes *literature, visual arts, performing arts*, and *music* as separate domains. For each domain, it includes three levels of involvement as described above (*interest, ability, performance*). For each sub-domain four questions were constructed. The first two questions refer to interest in a certain sub-domain. For example, for the “painting” sub-domain, “I have a strong interest in painting” and “I visit painting exhibitions.” The third question refers to ability, for example, “I paint pictures,” and the forth question refers to performance, for example, “I have already exhibited my pictures publicly” (see **Table S1** in the Supplementary Materials).

**FIGURE 1:**
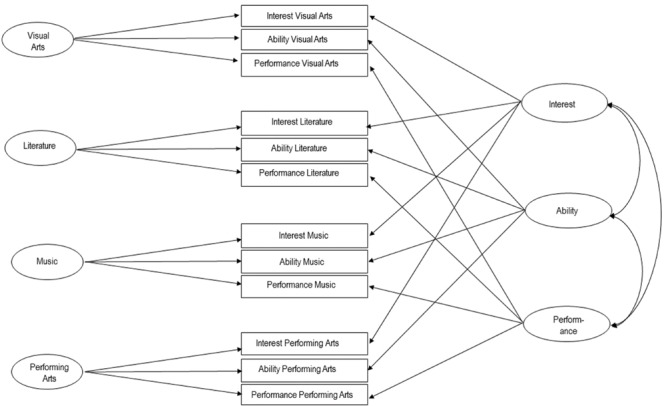
**Scale-structure of the Artistic Creativity Domains Compendium (ACDC)**.

For each scale mean scores were computed to provide a profile of the four domain-scales visual arts, literature, music and performing arts, the three levels of involvement and further the 12 scales of domain differentiated by level of involvement *interest* in *visual arts*/*literature/music/performing arts, ability* in *visual arts*/*literature/music/performing arts* and *performance* in *visual arts*/*literature/music/performing arts* (see **Table S1** in the Supplementary Materials). **Figure 1** shows the hypothesized scale structure.

### Participants

A total of 320 German speaking, healthy participants, 160 women and 160 men, aged 18–53 years (*M* = 26.19, *SD =* 8.52) were recruited from the general public. Approximately half of them were students, the other half had already completed their professional education. The study was approved by the institutional ethical review committee of the University of Bern. Participants signed written informed consent before data collection. They did not receive a reward for participation. Due to missing data, we had to eliminate 50 data sets. Thus, the final sample consisted of 270 participants.

### Materials

#### Artistic Creativity

The ACDC consists of 72 questions about interest, ability and performance in four artistic domains (visual arts, literature, music and performing arts) and 18 corresponding sub-domains (painting, sculpting, photography, graphic design, fictional-writing, poetry, play-writing, journalism, classical music, jazz music, rock music, folk music, movie-acting, theater-acting, dancing, ballet-dancing, musical performance). The full questionnaire is presented in **Table S1** in the Supplementary Materials. In this study we used a computerized version. For analysis, mean-scores were computed overall, across domains (*visual arts, literature, music* and *performing arts*), across levels of involvement (*interest, ability*, and *performance*), and across both domains and levels of involvement (*interest* in *visual arts, ability* in *visual arts, performance* in *visual arts*; *interest* in *literature, ability* in *literature, performance* in *literature*; *interest* in *music, ability* in *music, performance* in *music*; *interest* in *performing arts, ability* in *performing arts, performance* in *performing arts)*. It is available in German and in English, the present study was conducted with the German version.

#### Convergent Thinking

The *Remote Associates Test* (RAT) by Mednick and Mednick (1971) was used to assess convergent thinking (translated into German by Bolte et al., 2003). Thirty word triads were taken from the modified version. Triads consisted of nouns only. A total sum-score of all correct answers was calculated.

#### Divergent Thinking

In order to assess divergent thinking, the *Abbreviated Torrance Test for Adults* translated to German (ATTA, Goff and Torrance, 2002) and the *Sentence Construction* sub-test of the German *Analyse des Schlussfolgernden und Kreativen Denkens* (ASK, Schuler and Hell, 2005) were used.

The ATTA consists of one verbal and two figural tasks. In the verbal task, a fictional scenario is presented. Participants are instructed to imagine as many problems as possible that might occur in this situation. In the two figural tasks, the participants are presented with incomplete figures provided on a test sheet. They are instructed to complete them and to give a title for each picture. Two independent raters scored the tasks according to the manual (i.e., Fluency, Originality, Richness and Colourfulness of Imagery, Emotion/Feelings, Future Orientation, Humor and Provocativeness for the verbal task and Elaboration, Flexibility, Openness, Unusual Visualization, Movement/Sound, Richness and Colorfulness of Imagery, Abstractness of Titles, Articulateness, Combination of Figures, Internal Visual Perspective, Emotion and Fantasy for the figural task). Interrater-reliability for the present sample was *r* = 0.91. We computed a mean score of both ratings.

The ASK, consists of the presentation of four capital letters. Participants are instructed to construct four-word sentences with these letters as the initial letters. A sum score of all countable sentences in both trials was calculated and ranked according to the manual.

A *figural divergent thinking score* was calculated as the mean of all figural scales of the ATTA and a *verbal divergent thinking* score was calculated as the mean of all verbal scales of the ATTA and the sum score of the ASK.

#### Intelligence

The *Wortschatztest* (WST), a German vocabulary test was used to assess verbal intelligence (Schmidt and Metzler, 1992). The test consists of 42 words and 210 pseudo-words. Each trial consists of one word and five pseudo-words. The intelligence score was calculated as the total number of correct minus incorrect responses.

#### Attention

To measure attention, the D2-R was used (Brickenkamp et al., 2010). It consists of a sheet of paper that contains the letters *d* and *p* which are combined with different numbers of apostrophes. Participants have to find the letters *d* with two apostrophes and circle them as fast as possible. The number of correct detections per line were summarized, excluding the first and the last line, and false positives were subtracted. The results are used to calculate a sum score which is then transformed according to age norms.

#### Working Memory

Working memory was tested with a German version of the *Reading Span Task* (RST; Daneman and Carpenter, 1980, cf. Jaeggi et al., 2010). It consists of 100 unrelated sentences. Half of them make sense semantically and half do not but all are syntactically correct. Each sentence contains 6–15 words (*M*: 10.05; *SD*: 1.98) with a mean word length of 6.25 (*SD*: 0.81). Reading-span is scored as the set-size of the block in which all words of at least three sets can be remembered correctly or the block in which at least two sets were remembered minus 0.5.

### Procedure

After signing written informed consent, participants were tested individually. The study consisted of the ACDC, tests of divergent and convergent thinking, intelligence, working memory, and attention. The ordering of the tests is displayed in **Table 1**.

**Table 1:** Procedure: ordering of tasks.

Construct	Test	Time (minutes)
Attention	D2-R	8
Convergent thinking	RAT	6
Working memory	RST	25
Divergent figural thinking	ATTA	10
Divergent verbal thinking	ASK	7
Verbal intelligence	WST	13
Artistic creativity	ACDC	10

*For abbreviations see text.*

For the D2-R (Brickenkamp et al., 2010) participants were given a paper and pencil and were instructed to cross out all *d*-target-stimuli combined with two dashes, distributed in an exercise-line including 26 target-stimuli and 31 distractor-stimuli. The test consisted of 14 lines, which had to be filled out successively without break. Participants had to start a new line every 20 s. Next, the *RAT* was administered. Participants were instructed to find the single fourth word that could possibly connect the three former unrelated words of each triad. They solved the 30 word triads of the *RAT*, presented on one sheet, within 5 min (Bolte et al., 2003). Afterward, the *RST* was conducted (Daneman and Carpenter, 1980). Subjects were asked to read sentences aloud that were presented on a computer screen, decide whether they made sense or not and to memorize each last word of the sentence. Set-size ranged from two sentences to six sentences in a non-random order with increasing difficulty. The sentences were presented in the center of a white screen and participants pressed 1 if the sentence made sense and 0 if not. At the end of each set the instruction to recall the final words of each previously read-out sentence in the correct order appeared on a white screen. Responses were collected by the investigator. Five blocks of five sets each were presented consecutively.

Next, the divergent thinking tests were administered. In the verbal task of the ATTA (Goff and Torrance, 2002), participants were presented the situation that they are able to fly, on a sheet. They had 3 min to write down as many problems they thought could occur in this situation. In the figural task of the ATTA, participants received sheets with incomplete figures they had to turn into interesting drawings for which titles had to be invented. The participants had 3 min to complete each of the three tasks, starting with the verbal one, followed by the two figural tasks. Next, the ASK (Schuler and Hell, 2005) was administered as a further measure of verbal divergent thinking. Participants were twice presented four capital letters on a sheet and instructed to invent as many four-word-sentences as possible. They had three trials for both combinations. Then the WST (Schmidt and Metzler, 1992) was administered to test verbal intelligence. In 42 trials the participants were asked to each time choose the one existing word beside five distractor-pseudo-words. Words were presented in a 16-point font with an associated number between 1 and 6 each. Participants were instructed not to guess, but to press 0 if they did not know the answer. Answers could be given with the keyboard. As a last test, the ACDC was conducted on the computer. Participants answered the 72 questions with the keyboard on a four point Likert-Scale ranging from 1 = strongly disagree/never over 2 = disagree/rarely and 3 = agree/sometimes to 4 = strongly agree/frequently.

### Statistical Analysis

#### Analysis of the Structure of the ACDC

To analyze the scale-structure of the ACDC we used a *Multi-Trait-Multi-Methods-Model* (MTMM). This allows investigating artistic creativity from two view-points, the four different domains and the three different levels of involvement (Nussbeck et al., 2012). The sub-domain-scales – divided on levels of involvement – could thereby be explained by (besides the error components) the domains and the level of involvement (Eid, 2000). For robust results and in order to keep a minimum of 200 cases per analysis we applied bootstrapping. The MTMM was calculated with AMOS using asymptotically distribution free estimation to control for skewed distributions.

#### External Validity

Correlations were analyzed in SPSS with z-transformed data. Significance level was set to α = 0.05.

## Results

Mean and standard deviation for all variables are displayed in **Table 2**.

**Table 2:** Mean values and standard deviations for Artistic Creativity Domains Compendium scores, creativity tests, and cognitive tests.

Test-score	*M*	*SD*
ACDC total	1.71	0.30
Visual arts	1.90	0.47
Literature	1.51	0.32
Music	1.70	0.38
Performing arts	1.73	0.41
ACDC interest	2.14	0.44
ACDC ability	1.57	0.34
ACDC performance	1.16	0.18
Interest in visual arts	2.20	0.61
Ability in visual arts	2.03	0.62
Performance in visual arts	1.18	0.34
Interest in literature	1.98	0.55
Ability in literature	1.21	0.31
Performance in literature	1.08	0.16
Interest in music	2.17	0.49
Ability in music	1.47	0.54
Performance in music	1.15	0.26
Interest in performing arts	2.18	0.58
Ability in performing arts	1.62	0.52
Performance in performing arts	1.22	0.30
ASK	100.99	9.93
ATTA mean verbal score 1	1.58	1.32
ATTA mean figural score 1	6.29	2.73
ATTA mean verbal score 2	11.07	5.07
ATTA mean figural score 2	23.24	6.88
RAT	5.01	2.34
Verbal intelligence	31.16	3.69
Attention	105.69	9.57
Working memory	2.79	1.13

### Structure of the ACDC

**FIGURE 2:**
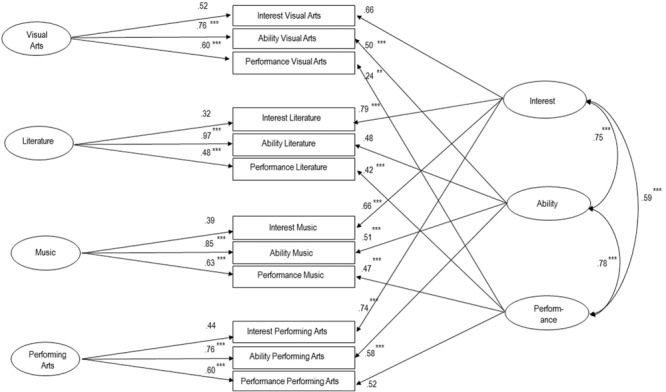
**Multi-Trait-Multi-Methods-Model (MTMM) of the Artistic-Creativity-Domains-Compendium (ACDC), standardized solution.** One-sided arrows: factor-loadings; two-sided arrows: correlations. ^∗∗^*p* < 0.01, ^∗∗∗^*p* < 0.001. Factor-loadings without asterisk have been set to 1 prior to estimation (unstandardized solution).

We generated an MTMM-model with 200 bootstraps according to the structure of the ACDC, that is, 72 items in four domains separated by level of involvement (interest, ability and performance in visual arts, literature, music and performing arts). The fit was good with χ^2^(39) = 55.75, *p* < 0.05, *Comparative Fit Index* (CFI) = 0.96, *Tucker Lewis Coefficient* (TLI) = 0.94, and a *root mean square error* (RMSEA) of 0.04. The indicator-reliabilities of the domains by levels of involvement were all above 0.4, indicating good convergent validity and implying that all 12 scales can be maintained as constructed. The complete model with standardized correlations and factor-loadings is presented in **Figure 2**.

### Internal Consistency of the ACDC

Internal consistency was high, with Cronbach’s alpha of α = 0.93 for the 72 items as a whole. For the four domains, internal reliability was α = 0.88 for *visual arts*, α = 0.80 for *literature*, α = 0.84 for *music* and α = 0.87 for *performing arts*.

For the levels of involvement resulted α = 0.91 for *interest*, α = 0.77 for *ability* and α = 0.68 for *performance*. Further differentiation of levels of involvement for each domain resulted in α = 0.84 for *interest* in *visual arts*, α = 0.71 for *ability* in *visual arts*, α = 0.57 for *performance* in *visual arts*. For *literature* resulted α = 0.77 for *interest*, α = 0.37 for *ability* and α = 0.25 for *performance*. For *interest* in *music* α = 0.75 was obtained, for *ability* in *music* resulted α = 0.75 and for *performance* in *music* α = 0.31. For *performing arts* resulted α = 0.83 for *interest*, α = 0.52 for *ability* and α = 0.59 for *performance*.

#### External Validity

In order to test the external validity of the ACDC, we assessed its relation to divergent and convergent thinking. The *figural divergent thinking* was computed as a mean from the scores ATTA Mean Figural 1 and 2, and *verbal divergent thinking* was computed from ATTA Mean Verbal Score 1 and 2 and ASK (cf. **Table 2**). The mean score of the ACDC correlated significantly with the *figural divergent thinking* score and the *verbal divergent thinking* score. The correlation with the *convergent thinking* score was not significant.

On the level of *involvement in art*, the *figural divergent thinking* score correlated with *interest, ability*, and *performance*. The *verbal divergent thinking* score also correlated significantly with *interest, ability*, and *performance*. Results for the *convergent* score of the RAT were not significant. These correlations are presented in **Table 3**.

**Table 3:** Correlations between the overall ACDC-Score, ACDC-levels, ACDC-domains and divergent and convergent thinking scores.

	ACDC T	ACDC I	ACDC A	ACDC S	ACDC VA	ACDC L	ACDC M	ACDC PA	DF	DV	CV
ACDC total	1										
ACDC interest	0.95**	1									
ACDC ability	0.86**	0.69**	1								
ACDC performance	0.70**	0.49**	0.70**	1							
Visual arts	0.74**	0.71**	0.63**	0.48**	1						
Literature	0.76**	0.76**	0.62**	0.46**	0.43**	1					
Music	0.74**	0.68**	0.72**	0.51**	0.37**	0.48**	1				
Performing arts	0.79**	0.74**	0.65**	0.64**	0.41**	0.53**	0.38**	1			
Divergent (Figural)	0.17**	0.16**	0.15*	0.12*	0.15*	0.07	0.05	0.22**	1		
Divergent (Verbal)	0.33**	0.27**	0.38**	0.25**	0.25**	0.30**	0.19**	0.28**	0.26**	1	
Convergent	-0.04	-0.05	-0.01	0.00	-0.02	-0.02	-0.02	-0.04	-0.01	0.06	1

*ACDC, Artistic Creativity Domains Compendium, ^∗^*p* < 0.05, ^∗∗^p < 0.01.*

On the level of *art domains*, the *divergent figural* mean-score was significantly correlated with *visual arts* and *performing arts*. The *divergent verbal* mean-score correlated significantly with *visual arts, literature, music* and *performing arts*. The *convergent* score of the RAT correlated with none of the domain scores of the ACDC. These correlations are likewise presented in **Table 3**.

The correlations between artistic domains divided further by levels of involvement are presented in **Table 4**. They showed a significant correlation for the *figural divergent thinking* score with *interest* in *visual arts, ability* in *visual arts, interest* in *performing arts, ability* in *performing arts*, and *performance* in *performing arts*. The *verbal divergent* thinking score correlated significantly with *interest* in *visual arts, ability* in *visual arts, interest* in *literature, ability* in *literature, performance* in *literature, ability* in *music, interest* in *performing arts, ability* in *performing arts*, and *performance* in *performing arts*. Convergent thinking correlated with none of the domain scores divided by levels of involvement of the ACDC.

**Table 4:** Correlations between ACDC domains divided by levels of involvement, divergent, and convergent thinking scores.

	I VA	A VA	P VA	I L	A L	P L	I M	A M	P M	I PA	A PA	P PA	DF	DV	CV
Interest in visual arts	1														
Ability in visual arts	0.64^∗∗^	1													
Performance in visual arts	0.40^∗∗^	0.54^∗∗^	1												
Interest in literature	0.50^∗∗^	0.30^∗∗^	0.11	1											
Ability in literature	0.27^∗∗^	0.29^∗∗^	0.13^∗^	0.63^∗∗^	1										
Performance in literature	0.17^∗∗^	0.22^∗∗^	0.16^∗∗^	0.40^∗∗^	0.67^∗∗^	1									
Interest in music	0.45^∗∗^	0.25^∗∗^	0.10	0.50^∗∗^	0.25^∗∗^	0.20^∗∗^	1								
Ability in music	0.21^∗∗^	0.25^∗∗^	0.19^∗∗^	0.39^∗∗^	0.31^∗∗^	0.26^∗∗^	0.56^∗∗^	1							
Performance in music	0.14^∗^	0.18^∗^	0.24^∗∗^	0.25^∗∗^	0.20^∗∗^	0.19^∗∗^	0.41^∗∗^	0.72^∗∗^	1						
Interest in performing arts	0.47^∗∗^	0.32^∗∗^	0.10	0.59^∗∗^	0.33^∗∗^	0.30^∗∗^	0.42^∗∗^	0.27^∗∗^	0.23^∗∗^	1					
Ability in performing arts	0.26^∗∗^	0.28^∗∗^	0.07	0.32^∗∗^	0.25^∗∗^	0.28^∗∗^	0.21^∗∗^	0.25^∗∗^	0.18^∗∗^	0.69^∗∗^	1				
Performance in performing arts	0.24^∗∗^	0.23^∗∗^	0.21^∗∗^	0.29^∗∗^	0.29^∗∗^	0.37^∗∗^	0.18^∗∗^	0.25^∗∗^	0.31^∗∗^	0.52^∗∗^	0.67^∗∗^	1			
Divergent (Figural)	0.15^∗^	0.14^∗∗^	0.05	0.06	0.05	0.07	0.06	0.03	0.02	0.21^∗∗^	0.21^∗∗^	0.15^∗∗^	1		
Divergent (Verbal)	0.21^∗^	0.27^∗∗^	0.11	0.28^∗∗^	0.23^∗∗^	0.20^∗∗^	0.12	0.25^∗∗^	0.11	0.24^∗∗^	0.27^∗∗^	0.23^∗∗^	0.26^∗∗^	1	
Convergent	-0.04	0.09	0.02	-0.01	-0.05	0.00	-0.06	0.02	0.02	-0.04	-0.04	-0.02	-0.01	0.06	1

*ACDC, Artistic Creativity Domains Compendium, ^∗^*p* < 0.05, ^∗∗^*p* < 0.01.*

### ACDC and Cognition

The total score of the ACDC correlated with *verbal intelligence*. On level of involvement, *verbal intelligence* correlated with *interest*, and *ability*. *Attention* and *working memory* did not correlate with any of the levels of involvement. Correlations are presented in **Table 5**.

**Table 5:** Correlations between the overall ACDC-Score, ACDC-levels, ACDC-domains and cognitive functions.

	ACDC T	ACDC I	ACDC A	ACDC S	ACDC VA	ACDC L	ACDC M	ACDC PA	VI	A	WM
ACDC total	1										
ACDC interest	0.95^∗∗^	1									
ACDC ability	0.86^∗∗^	0.69^∗∗^	1								
ACDC performance	0.70^∗∗^	0.49^∗∗^	0.70^∗∗^	1							
Visual arts	0.74^∗∗^	0.71^∗∗^	0.63^∗∗^	0.48^∗∗^	1						
Literature	0.76^∗∗^	0.76^∗∗^	0.62^∗∗^	0.46^∗∗^	0.43^∗∗^	1					
Music	0.74^∗∗^	0.68^∗∗^	0.72^∗∗^	0.51^∗∗^	0.37^∗∗^	0.48^∗∗^	1				
Performing arts	0.79^∗∗^	0.74^∗∗^	0.65^∗∗^	0.64^∗∗^	0.41^∗∗^	0.53^∗∗^	0.38^∗∗^	1			
Verbal intelligence	0.25^∗∗^	0.27^∗∗^	0.19^∗∗^	0.09	0.15^∗^	0.27^∗∗^	0.23^∗∗^	0.13^∗^	1		
Attention	-0.00	-0.03	0.04	0.01	-0.11	-0.06	-0.04	0.16^∗∗^	0.15^∗^	1	
Working memory	0.03	-0.02	0.11	0.08	-0.04	0.14^∗∗^	0.02	0.02	0.22^∗∗^	0.10	1

*ACDC, Artistic Creativity Domains Compendium, ^∗^*p* < 0.05, ^∗∗^*p* < 0.01.*

We also explored the relation between specific artistic domains and intelligence, attention, and working memory. On level of domains, *verbal intelligence* correlated with *visual arts, literature, music* and *performing arts. Attention* correlated with *performing arts. Working memory* did correlate with *literature*. All correlations are likewise shown in **Table 5**.

Further, for the artistic domains of the ACDC divided by levels of involvement *Ability* in *performing arts* correlated positively with *attention*. *Interest* in *visual arts, interest* in *literature, interest* in *music, interest* in *performance* and *ability* in *literature, ability* in *music* and *performance* in *music* correlated with *verbal intelligence*. *Interest* in *literature* and *ability* in *literature* and *performance* in *music* correlated with *working memory.* **Table 6** shows all correlations.

**Table 6:** Correlations between ACDC domains divided by levels of involvement, divergent, and convergent thinking scores.

	I VA	A VA	P VA	I L	A L	P L	I M	A M	P M	I PA	A PA	P PA	VI	A	WM
Interest in visual arts	1														
Ability in visual arts	0.64^∗∗^	1													
Performance in visual arts	0.40^∗∗^	0.54^∗∗^	1												
Interest in literature	0.50^∗∗^	0.30^∗∗^	0.11	1											
Ability in literature	0.27^∗∗^	0.29^∗∗^	0.13^∗^	0.63^∗∗^	1										
Performance in literature	0.17^∗∗^	0.22^∗∗^	0.16^∗∗^	0.40^∗∗^	0.67^∗∗^	1									
Interest in music	0.45^∗∗^	0.25^∗∗^	0.10	0.50^∗∗^	0.25^∗∗^	0.20^∗∗^	1								
Ability in music	0.21^∗∗^	0.25^∗∗^	0.19^∗∗^	0.39^∗∗^	0.31^∗∗^	0.26^∗∗^	0.56^∗∗^	1							
Performance in music	0.14^∗^	0.18^∗^	0.24^∗∗^	0.25^∗∗^	0.20^∗∗^	0.19^∗∗^	0.41^∗∗^	0.72^∗∗^	1						
Interest in performing arts	0.47^∗∗^	0.32^∗∗^	0.10	0.59^∗∗^	0.33^∗∗^	0.30^∗∗^	0.42^∗∗^	0.27^∗∗^	0.23^∗∗^	1					
Ability in performing arts	0.26^∗∗^	0.28^∗∗^	0.07	0.32^∗∗^	0.25^∗∗^	0.28^∗∗^	0.21^∗∗^	0.25^∗∗^	0.18^∗∗^	0.69^∗∗^	1				
Performance in performing arts	0.24^∗∗^	0.23^∗∗^	0.21^∗∗^	0.29^∗∗^	0.29^∗∗^	0.37^∗∗^	0.18^∗∗^	0.25^∗∗^	0.31^∗∗^	0.52^∗∗^	0.67^∗∗^	1			
Verbal intelligence	0.21^∗∗^	0.05	-0.01	0.30^∗∗^	0.14^∗^	0.08	0.19^∗∗^	0.22^∗∗^	0.15^∗^	0.15^∗^	0.09	0.05	1		
Attention	-0.12	-0.06	-0.07	-0.05	-0.10	-0.01	-0.06	0.01	-0.06	0.12	0.22^∗∗^	0.11	0.15^∗^	1	
Working memory	-0.08	0.04	-0.01	0.12^∗^	0.17^∗∗^	0.04	-0.08	0.11	0.15^∗^	-0.00	0.03	0.04	0.22^∗∗^	0.10	1

*ACDC, Artistic Creativity Domains Compendium, ^∗^*p* < 0.05, ^∗∗^*p* < 0.01.*

Finally, we also analyzed the relationship between *divergent* and *convergent thinking, verbal intelligence, attention*, and *working memory*. The *figural divergent thinking* score correlated with *attention*. The *verbal divergent thinking* score correlated with *verbal intelligence* and *working memory*. The *convergent thinking* score correlated significantly with *verbal intelligence*. These correlations are shown in **Table 7**.

**Table 7:** Correlations between divergent and convergent thinking, intelligence, attention, and working memory.

	DF	DV	CV	Intelligence	Attention	WM
Divergent (Figural)	1					
Divergent (Verbal)	0.26^∗∗^	1				
Convergent	-0.01	0.06	1			
Verbal intelligence	0.07	0.32^∗∗^	0.20^∗∗^	1		
Attention	0.13^∗^	0.12	0.01	0.15^∗^	1	
Working memory	0.05	0.19^∗∗^	0.06	0.22^∗∗^	0.10	1

*^∗^*p* < 0.05, ^∗∗^*p* < 0.01.*

## Discussion

We present the ACDC, a new questionnaire that covers artistic creativity in different domains (*visual arts, literature, music* and *performing arts)* on different levels of involvement (*interest in, ability to*, and *performance)*. We used the ACDC to investigate the relation between domains, sub-domains and levels of involvement and cognitive functions in a differentiated way. Internal consistency among the four domains as well as the levels of involvement was very good. The MTMM-model that was used to separate the domains and levels of involvement showed a good fit. It supports a model of artistic creativity that differentiates levels of involvement for each sub-domain. Moreover, the good fit of the model structure with uncorrelated domains of artistic creativity suggests a clear specificity of the scales. The indicator-reliabilities of the sub-scales were high and the factor loadings for subordinate scales were mostly high, supporting the scale-construction of the questionnaire. However, lower loadings on some sub-scales in the domains of literature and music, for instance, may reflect the skewed distribution that is typical for this kind of non-expert population (Silvia et al., 2012). In another population, for instance for a sample of authors or musicians, these items might be more selective. These results together with a good internal consistency of each of the domain scales and the total score support the necessity to assess them separately. Low factor loadings, for example in the domain *literature* are supposedly due to low variance in that particular scale. The correlations between levels of involvement are very high. However, the by far lowest correlation between interest and performance still supports our suggestion to observe the scales separately.

In the external validation of the ACDC the correlations support the hypothesis that it indeed measures forms of divergent creativity. The non-significant correlation between the ACDC and convergent thinking might suggest that artistic creativity is rather related to divergent than to convergent thinking. In future studies, tests of figural convergent creativity should be included to see if the results are similar.

On the level of artistic domains, *visual arts* and *performing arts* correlate significantly with the *divergent figural* mean-score whereas *music* and *literature* did not. These results demonstrate that the ACDC does not only assess general divergent thinking but also shows differences between the domains. Moreover, the higher correlation with *performing arts* indicates that this domain shares a higher portion of divergent figural creativity. This complements earlier findings in which physical activity was strongly correlated with higher divergent creativity (Best, 2010). To exclude the possibility that the higher correlation is due to higher physical activity in people who practice *performing arts*, it is therefore important to control for an influence of general physical activity.

The significant correlation between each of the domains *visual arts, music, literature*, and *performing arts* of the ACDC and the *divergent verbal* mean-score, with *literature* correlating highest, suggests that the domain *literature* might share the highest portion of verbal divergent thinking. On level of involvement, the ACDC levels *interest, ability*, and *performance* correlated significantly with *figural* and *verbal divergent thinking.* For verbal divergent thinking, *ability*, as assessed with the ACDC, showed a higher correlation. This result indicates that the divergent verbal tests represent divergent creative thinking best on the medium level of involvement.

In sum, the external validation of the ACDC with divergent and convergent tests indicates that the questionnaire measures the construct “artistic creativity” that correlates with divergent creativity and does not overlap with convergent creativity. Moreover, the differing results among domains and levels of involvement concerning figural and verbal scores support the expected separation of domains and levels of involvement.

Further, the analysis of the correlations between the ACDC, intelligence, attention and working memory also showed interesting results. Only *verbal intelligence* correlated significantly with the ACDC sum score together with all four domains *visual arts, literature, music* and *performing arts* on domain-level. These results indicate a relation between artistic creativity and intelligence that is not domain specific. A relationship between musicality and intelligence has been investigated by previous studies (Schellenberg, 2004; Moreno et al., 2011). The division of domains by level of involvement shows that on level of *ability* or *performance* only *ability* in *music* and *literature* and *performance* in *music* correlate significantly with *verbal intelligence*. On level of involvement *interest* and *ability* correlated significantly with the *verbal intelligence*, with *interest* correlating higher than *ability.* On level of *interest, interest* in *visual arts, literature, music* and *performing arts* correlated with *verbal intelligence*. These results, showing that interest in several domains is related to *verbal intelligence*, complement findings that people scoring high on openness to experience also show higher intelligence scores (Silvia and Sanders, 2010).

*Attention* correlated significantly positive with *performing arts* overall as well as *ability* in *performing arts.* This indicates the importance of a differentiated assessment of forms, domains and sub-domains of creativity and anticipate different processes for them. The fact that *ability* in *performing arts* correlated significantly with attention gives interesting insights into potential mechanisms of this domain. Here, action taking might be important. Attention could play a crucial role in the execution of *performing arts* like dance and play. It remains to clarify if the effects arise from a higher proportion of physical activity in general as described before concerning *performing arts* and divergent creativity (Best, 2010). Future studies should control with questions about weekly physical activity. The negative correlation of *visual arts* with attention sheds further light on the differing former results concerning its relation with divergent verbal and figural and artistic thinking.

Working memory correlated with *literature* overall and with *interest* and *ability* in *literature.* As the RST is a verbal working memory task future studies should relate the ACDC to non-verbal tests of working memory.

To further analyze the correlations between artistic creativity and cognitive functioning we compared them to the correlations obtained between divergent and convergent tests and cognitive functioning. The divergent figural creative test correlated positively with attention whereas the verbal test did not. The fact that both parties also significantly correlate with *performing arts*, lead to hypothesize a domain specific positive relation between divergent figural creativity and attention, specifically triggered by *performing arts.* Only the divergent verbal task on the other hand, correlated with *verbal intelligence* and the working memory task. This seems to provide evidence that divergent verbal and figural creative tasks and the different ACDC scales do not exclusively measure the same share of creativity. Moreover, it leads to the question if other forms of creativity, for example scientific creativity, would correlate differently with measures of working memory. Future studies that aim to investigate other forms of creativity could shed light on this question. A*s* the RST is a verbal measurement of working memory, a different pattern of relationships may emerge for figural or numerical working memory tasks. Same applies for measures of intelligence. In future studies, figural intelligence tests could also be included to clarify if the relation between artistic creativity and intelligence is specific for *verbal intelligence*.

## Conclusion

The ACDC is a new easy to use questionnaire that enables to assess artistic creativity in several domains and sub-domains. It provides separate scales for interest, ability, and performance, providing for fine-grained results. Moreover, the ACDC offers the possibility to study changes across development, in training studies, or to follow up on pathological changes. It also gives the opportunity to investigate relationships between different aspects of artistic creativity and personality traits, affective, or cognitive style in a straight-forward way. Further, our results show that relationships between creativity and cognitive functioning are most pronounced within domains and at the level of interest and ability. They show that different domains and sub-domains build on different cognitive functions. Interestingly all four domains of artistic creativity, on a level of interest, rather relate to more complex cognitive functions like *verbal intelligence*, than to basic cognitive functions like attention. This is important for future research in order to disentangle the relationships between different domains of creativity and general aspects of cognition.

## Author Contributions

All authors listed, have made substantial, direct and intellectual contribution to the work, and approved it for publication.

## Conflict of Interest Statement

The authors declare that the research was conducted in the absence of any commercial or financial relationships that could be construed as a potential conflict of interest.

## Supplementary Material

TABLE S1 **Artistic Creativity Domains Compendium (ACDC)**.

IMAGE 1 **ACDC-Profile**.
