# Supplementary material for: Disentangling the Impact of Artistic Creativity on Creative Thinking, Working Memory, Attention, and Intelligence: Evidence for Domain-Specific Relationships with a New Self-Report Questionnaire
Source: Front Psychol. 2016 Jul 28;7:1089. doi: 10.3389/fpsyg.2016.01089 (PMC4963383; doi:10.3389/fpsyg.2016.01089)
Supplement: TABLE S1 — Artistic Creativity Domains Compendium (ACDC). [file Table_1.DOCX]

Artistic Creativity Domains Compendium (ACDC)
(Meier, 2014)

| 1. Ich interessiere mich sehr für Malerei | stimmt überhaupt nicht | stimmt eher nicht | stimmt eher | stimmt vollkommen |
| --- | --- | --- | --- | --- |
| 2. Ich habe schon eine Bilderausstellung besucht | nie | selten | ab und zu | häufig |
| 3. Ich habe schon selbst ein Bild gemalt | nie | selten | ab und zu | häufig |
| 4. Ich habe eigene Bilder schon öffentlich ausgestellt | nie | selten | ab und zu | häufig |
| 5. Ich interessiere mich sehr für Skulpturen | stimmt überhaupt nicht | stimmt eher nicht | stimmt eher | stimmt vollkommen |
| 6. Ich habe schon eine Skulpturenausstellung besucht | nie | selten | ab und zu | häufig |
| 7. Ich habe schon selbst eine Skulptur angefertigt | nie | selten | ab und zu | häufig |
| 8. Ich habe schon eigene Skulpturen öffentlich ausgestellt | nie | selten | ab und zu | häufig |
| 9. Ich interessiere mich sehr für Photographie | stimmt überhaupt nicht | stimmt eher nicht | stimmt eher | stimmt vollkommen |
| 10. Ich habe schon eine Fotoausstellung besucht | nie | selten | ab und zu | häufig |
| 11. Ich habe schon selbst künstlerische Fotografien aufgenommen | nie | selten | ab und zu | häufig |
| 12. Ich habe schon eigene Fotografien öffentlich ausgestellt | nie | selten | ab und zu | häufig |
| 13. Ich interessiere mich sehr für Grafikdesign | stimmt überhaupt nicht | stimmt eher nicht | stimmt eher | stimmt vollkommen |
| 14. Ich habe schon eine Grafikausstellung besucht | nie | selten | ab und zu | häufig |
| 15. Ich habe schon selbst ein grafisches Kunstwerk (z.B. ein Plakat) angefertigt | nie | selten | ab und zu | häufig |
| 16. Ich habe eigene Grafiken schon öffentlich ausgestellt | nie | selten | ab und zu | häufig |
| 17. Ich interessiere mich sehr für Literatur | stimmt überhaupt nicht | stimmt eher nicht | stimmt eher | stimmt vollkommen |
| 18. Ich habe schon selbst einen Roman zu schreiben begonnen, aber nicht fertiggestellt | nie | selten | ab und zu | häufig |
| 19. Ich habe schon selbst einen Roman geschrieben | nie | selten | ab und zu | häufig |
| 20. Ich habe schon einen eigenen Roman veröffentlicht | nie | selten | ab und zu | häufig |
| 21. Ich interessiere mich sehr für Poesie | stimmt überhaupt nicht | stimmt eher nicht | stimmt eher | stimmt vollkommen |
| 22. Ich habe schon ein Gedicht für mich selbst geschrieben | nie | selten | ab und zu | häufig |
| 23. Ich habe schon ein Gedicht geschrieben und öffentlich vorgetragen | nie | selten | ab und zu | häufig |
| 24. Ich habe schon eigene Gedichte veröffentlicht | nie | selten | ab und zu | häufig |
| 25. Ich interessiere mich sehr für Theater und Schauspiel | stimmt überhaupt nicht | stimmt eher nicht | stimmt eher | stimmt vollkommen |
| 26. Ich habe schon selbst ein Theaterstück geschrieben | nie | selten | ab und zu | häufig |
| 27. Ich habe schon selbst ein Theaterstück geschrieben und öffentlich aufgeführt | nie | selten | ab und zu | häufig |
| 28. Ich habe schon ein Theaterstück geschrieben, das von jemand anderem öffentlich aufgeführt wurde | nie | selten | ab und zu | häufig |
| 29. Ich interessiere mich sehr für Journalismus | stimmt überhaupt nicht | stimmt eher nicht | stimmt eher | stimmt vollkommen |
| 30. Ich habe schon selbst einen Kommentar für eine Zeitschrift geschrieben | nie | selten | ab und zu | häufig |
| 31. Ich habe schon selbst einen Artikel für eine Zeitschrift geschrieben | nie | selten | ab und zu | häufig |
| 32. Ich habe schon eine Reportage für eine Zeitschrift geschrieben | nie | selten | ab und zu | häufig |
| 33. Ich interessiere mich sehr für Musik | stimmt überhaupt nicht | stimmt eher nicht | stimmt eher | stimmt vollkommen |
| 34. Ich habe schon selbst ein Musikstück komponiert | nie | selten | ab und zu | häufig |
| 35. Ich habe schon selbst einen Liedtext komponiert | nie | selten | ab und zu | häufig |
| 36. Von mir komponierte Musik wurde schon im Radio gespielt | nie | selten | ab und zu | häufig |
| 37. Ich interessiere mich sehr für klassische Musik | stimmt überhaupt nicht | stimmt eher nicht | stimmt eher | stimmt vollkommen |
| 38. Ich habe schon ein klassisches Konzert besucht | nie | selten | ab und zu | häufig |
| 39. Ich habe schon selbst klassische Musik gemacht | nie | selten | ab und zu | häufig |
| 40. Ich bin schon bei einem klassischen Konzert öffentlich aufgetreten | nie | selten | ab und zu | häufig |
| 41. Ich interessiere mich sehr für Jazzmusik | stimmt überhaupt nicht | stimmt eher nicht | stimmt eher | stimmt vollkommen |
| 42. Ich habe schon ein Jazzkonzert besucht | nie | selten | ab und zu | häufig |
| 43. Ich habe schon selbst Jazz gemacht | nie | selten | ab und zu | häufig |
| 44. Ich bin schon selbst bei einem Jazzkonzert öffentlich aufgetreten | nie | selten | ab und zu | häufig |
| 45. Ich interessiere mich sehr für Rockmusik | stimmt überhaupt nicht | stimmt eher nicht | stimmt eher | stimmt vollkommen |
| 46. Ich habe schon ein Rockkonzert besucht | nie | selten | ab und zu | häufig |
| 47. Ich habe schon selbst Rockmusik gemacht | nie | selten | ab und zu | häufig |
| 48. Ich bin schon bei einem Rockkonzert öffentlich aufgetreten | nie | selten | ab und zu | häufig |
| 49. Ich interessiere mich sehr für volkstümliche Musik | stimmt überhaupt nicht | stimmt eher nicht | stimmt eher | stimmt vollkommen |
| 50. Ich habe schon ein Volksmusikkonzert besucht | nie | selten | ab und zu | häufig |
| 51. Ich habe schon selbst Volksmusik gemacht | nie | selten | ab und zu | häufig |
| 52. Ich bin schon bei einem Volksmusikkonzert öffentlich aufgetreten | nie | selten | ab und zu | häufig |
| 53. Ich interessiere mich sehr für Schauspielerei | stimmt überhaupt nicht | stimmt eher nicht | stimmt eher | stimmt vollkommen |
| 54. Ich habe schon bei einer öffentlichen Theateraufführung eine Statistenrolle gespielt | nie | selten | ab und zu | häufig |
| 55. Ich habe schon bei einer öffentlichen Theateraufführung eine Nebenrolle gespielt | nie | selten | ab und zu | häufig |
| 56. Ich habe schon bei einer öffentlichen Theateraufführung eine Hauptrolle gespielt | nie | selten | ab und zu | häufig |
| 57. Ich interessiere mich sehr für Filme | stimmt überhaupt nicht | stimmt eher nicht | stimmt eher | stimmt vollkommen |
| 58. Ich habe schon selbst einen Film gedreht, der öffentlich aufgeführt wurde | nie | selten | ab und zu | häufig |
| 59. Ich habe schon selbst eine Nebenrolle gespielt in einem Film, der öffentlich ausgestrahlt wurde | nie | selten | ab und zu | häufig |
| 60. Ich habe schon selbst eine Hauptrolle gespielt in einem Film, der öffentlich ausgestrahlt wurde | nie | selten | ab und zu | häufig |
| 61. Ich interessiere mich sehr für Tanz | stimmt überhaupt nicht | stimmt eher nicht | stimmt eher | stimmt vollkommen |
| 62. Ich habe schon eine Tanzaufführung besucht | nie | selten | ab und zu | häufig |
| 63. Ich habe schon selbst getanzt | nie | selten | ab und zu | häufig |
| 64. Ich bin schon bei einer Tanzaufführung öffentlich aufgetreten | nie | selten | ab und zu | häufig |
| 65. Ich interessiere mich sehr für Ballett | stimmt überhaupt nicht | stimmt eher nicht | stimmt eher | stimmt vollkommen |
| 66. Ich habe schon eine Ballettaufführung besucht | nie | selten | ab und zu | häufig |
| 67. Ich habe schon selbst Ballett getanzt | nie | selten | ab und zu | häufig |
| 68. Ich bin schon bei einer Ballettaufführungen öffentlich aufgetreten | nie | selten | ab und zu | häufig |
| 69. Ich interessiere mich sehr für Musical | stimmt überhaupt nicht | stimmt eher nicht | stimmt eher | stimmt vollkommen |
| 70. Ich habe schon ein Musical besucht | nie | selten | ab und zu | häufig |
| 71. Ich habe schon selbst in einem Musical mitgewirkt | nie | selten | ab und zu | häufig |
| 72. Ich habe schon in einem Musical eine Hauptrolle gespielt | nie | selten | ab und zu | häufig |

Artistic Creativity Domains Compendium (ACDC)
(English translation)

| 1. I have a strong interest in painting. | strongly disagree | disagree | agree | strongly agree |
| --- | --- | --- | --- | --- |
| 2. I visit painting exhibitions. | never | rarely | sometimes | frequently |
| 3. I paint pictures. | never | rarely | sometimes | frequently |
| 4. I have already exhibited my pictures publicly. | never | rarely | sometimes | frequently |
| 5. I have a strong interest in sculptures. | strongly disagree | disagree | agree | strongly agree |
| 6. I visit sculpture exhibitions. | never | rarely | sometimes | frequently |
| 7. I make a sculpture myself. | never | rarely | sometimes | frequently |
| 8. I have already exhibited my sculptures publicly. | never | rarely | sometimes | frequently |
| 9. I have a strong interest in photography. | strongly disagree | disagree | agree | strongly agree |
| 10. I visit photo exhibitions. | never | rarely | sometimes | frequently |
| 11. I make artistic photos myself. | never | rarely | sometimes | frequently |
| 12. I have already exhibited my photos publicly. | never | rarely | sometimes | frequently |
| 13. I have a strong interest in graphic design. | strongly disagree | disagree | agree | strongly agree |
| 14. I visit graphic design exhibitions. | never | rarely | sometimes | frequently |
| 15. I create graphic design art (e.g. posters) myself. | never | rarely | sometimes | frequently |
| 16. I have already exhibited my graphic design art publicly. | never | rarely | sometimes | frequently |
| 17. I have a strong interest in literature. | strongly disagree | disagree | agree | strongly agree |
| 18. I already tried to write fiction myself but I did not finish it. | never | rarely | sometimes | frequently |
| 19. I write fiction myself. | never | rarely | sometimes | frequently |
| 20. I already published my fiction. | never | rarely | sometimes | frequently |
| 21. I have a strong interest in poetry. | strongly disagree | disagree | agree | strongly agree |
| 22. I write poems for myself. | never | rarely | sometimes | frequently |
| 23. I have already written a poem and recited it publicly. | never | rarely | sometimes | frequently |
| 24. I have already published poems. | never | rarely | sometimes | frequently |
| 25. I have a strong interest in theatre and play writing. | strongly disagree | disagree | agree | strongly agree |
| 26. I write plays myself. | never | rarely | sometimes | frequently |
| 27. I have already written a play and performed it publicly. | never | rarely | sometimes | frequently |
| 28. I have already written a play that was directed and performed by someone else publicly. | never | rarely | sometimes | frequently |
| 29. I have a strong interest in journalism. | strongly disagree | disagree | agree | strongly agree |
| 30. I write comments for journals. | never | rarely | sometimes | frequently |
| 31. I write articles for journals. | never | rarely | sometimes | frequently |
| 32. I write reportages for journals. | never | rarely | sometimes | frequently |
| 33. I have a strong interest in music. | strongly disagree | disagree | agree | strongly agree |
| 34. I compose music myself. | never | rarely | sometimes | frequently |
| 35. I compose lyrics myself. | never | rarely | sometimes | frequently |
| 36. Music I composed was already played in the radio. | never | rarely | sometimes | frequently |
| 37. I have a strong interest in classical music. | strongly disagree | disagree | agree | strongly agree |
| 38. I visit classical music concerts. | never | rarely | sometimes | frequently |
| 39. I play classical music myself. | never | rarely | sometimes | frequently |
| 40. I perform classical music publicly. | never | rarely | sometimes | frequently |
| 41. I have a strong interest in jazz. | strongly disagree | disagree | agree | strongly agree |
| 42. I visit jazz concerts. | never | rarely | sometimes | frequently |
| 43. I play jazz myself. | never | rarely | sometimes | frequently |
| 44. I perform jazz music publicly. | never | rarely | sometimes | frequently |
| 45. I have a strong interest in rock music. | strongly disagree | disagree | agree | strongly agree |
| 46. I visit rock concerts. | never | rarely | sometimes | frequently |
| 47. I play rock music myself. | never | rarely | sometimes | frequently |
| 48. I perform rock music publicly. | never | rarely | sometimes | frequently |
| 49. I have a strong interest in folk music. | strongly disagree | disagree | agree | strongly agree |
| 50. I visit a folk music concerts. | never | rarely | sometimes | frequently |
| 51. I play folk music myself. | never | rarely | sometimes | frequently |
| 52. I perform folk music publicly. | never | rarely | sometimes | frequently |
| 53. I have a strong interest in acting. | strongly disagree | disagree | agree | strongly agree |
| 54. I act in public plays as a background actor. | never | rarely | sometimes | frequently |
| 55. I act in public plays as a supporting actor. | never | rarely | sometimes | frequently |
| 56. I act in public plays as a main character. | never | rarely | sometimes | frequently |
| 57. I have a strong interest in movies. | strongly disagree | disagree | agree | strongly agree |
| 58. I record movies that are shown publicly. | never | rarely | sometimes | frequently |
| 59. I act as supporting character in movies that are shown publicly. | never | rarely | sometimes | frequently |
| 60. I act as main character in movies that are shown publicly. | never | rarely | sometimes | frequently |
| 61. I have a strong interest in dance. | strongly disagree | disagree | agree | strongly agree |
| 62. I visit dance performances. | never | rarely | sometimes | frequently |
| 63. I am a dancer myself. | never | rarely | sometimes | frequently |
| 64. I performed dance in public. | never | rarely | sometimes | frequently |
| 65. I have a strong interest in ballet. | strongly disagree | disagree | agree | strongly agree |
| 66. I visit ballet performances. | never | rarely | sometimes | frequently |
| 67. I am a ballet dancer myself. | never | rarely | sometimes | frequently |
| 68. I performed ballet in public. | never | rarely | sometimes | frequently |
| 69. I have a strong interest in musicals. | strongly disagree | disagree | agree | strongly agree |
| 70. I visit musicals. | never | rarely | sometimes | frequently |
| 71. I act in musicals as supporting actor. | never | rarely | sometimes | frequently |
| 72. I act in musicals as a main character. | never | rarely | sometimes | frequently |
