# Supplementary figures and images for: Disentangling the Impact of Artistic Creativity on Creative Thinking, Working Memory, Attention, and Intelligence: Evidence for Domain-Specific Relationships with a New Self-Report Questionnaire
Source: Front Psychol. 2016 Jul 28;7:1089. doi: 10.3389/fpsyg.2016.01089 (PMC4963383; doi:10.3389/fpsyg.2016.01089)

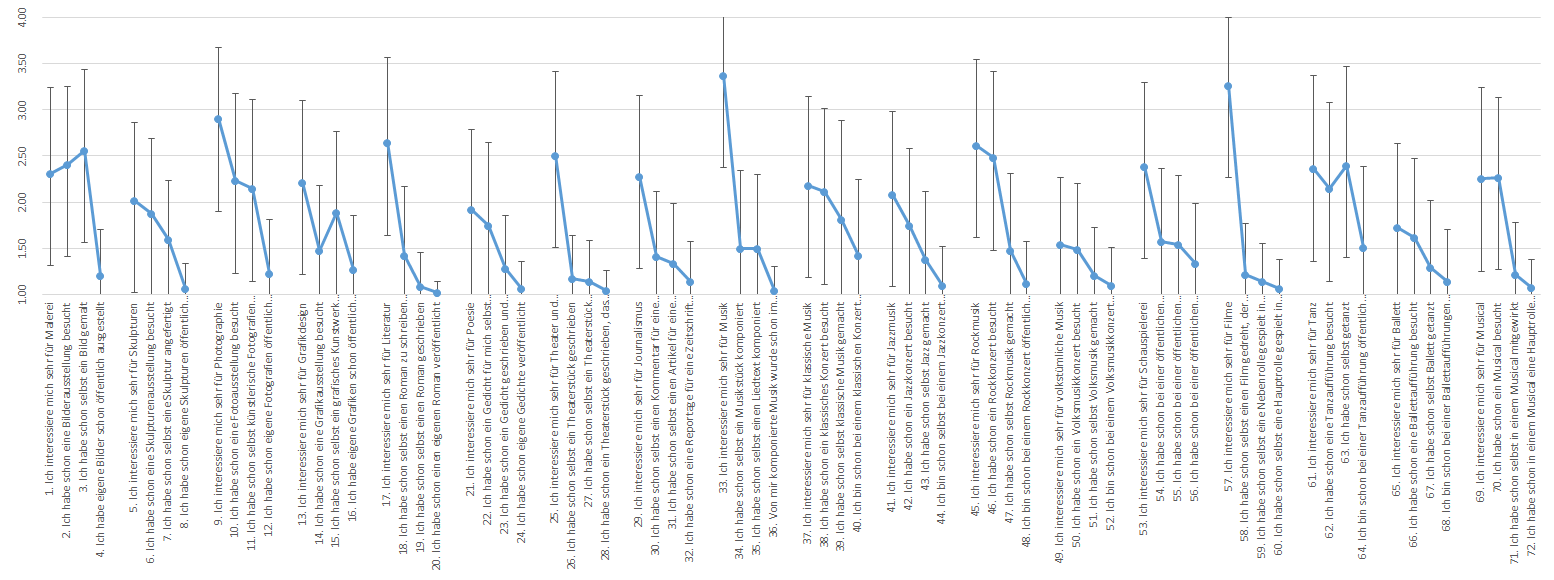

Supplement: IMAGE 1 — ACDC-Profile. [file Image_1.TIF]
